# A CRISPR-p53 interactome with potential implications for clinical CRISPR/Cas9 use

**DOI:** 10.18632/oncoscience.557

**Published:** 2022-05-09

**Authors:** Long Jiang, Fredrik Wermeling

**Affiliations:** ^1^Department of Medicine Solna, Center for Molecular Medicine, Karolinska University Hospital and Karolinska Institutet, Stockholm, Sweden

**Keywords:** CRISPR, p53, gene therapy, DNA damage response, cancer

## Abstract

CRISPR/Cas9-based tools are anticipated to transform the gene therapy field by facilitating the correction of disease-causing mutations. However, CRISPR/Cas9 generates DNA damage, which triggers a DNA damage response centered around the tumor-suppressor p53. In this research perspective, we discuss implications of this and describe a CRISPR-p53 interactome with cancer-related genes that, if mutated, can give cells a selective advantage following exposure to CRISPR/Cas9. We propose that the genes in the CRISPR-p53 interactome should be monitored in the clinical setting and describe that transient p53 inhibition could be used to limit the enrichment of cells with such mutations.

The development of CRISPR/Cas-based molecular biology tools represents a significant breakthrough for the gene therapy field [1]. As a testament to this, multiple clinical trials are ongoing using CRISPR/Cas9 to correct mutations causing various genetic diseases, for example in patients with sickle cell anemia and beta-Thalassemia [2, 3].

CRISPR/Cas-based tools can be used in a multitude of ways [4]. In the gene therapy setting, the currently used CRISPR/Cas approaches take advantage of its ability to generate double-stranded DNA (dsDNA) breaks at precise genetic locations. In this, a single guide RNA (sgRNA) is designed to bind the genetic region of interest, and is then delivered into the relevant cells together with an endonuclease, like Cas9, together forming a target-specific endonuclease complex. The resulting dsDNA breaks subsequently facilitate the introduction of genetic changes at the target site, e.g., correcting a disease-causing mutation.

Importantly, dsDNA breaks activate a cellular stress response, which can culminate in cell cycle arrest and apoptosis, arguably counteracting the intended outcome in the gene therapy setting. Central to this response is the tumor-suppressor protein p53, responsible for the transcription of a large set of genes linked to e.g. cell cycle arrest and apoptosis [5]. The critical role of p53 to limit cancer development is shown by that more than 50% of all cancers have inactivating mutations in *TP53* (the gene encoding for p53 in human) and that congenital mutations in *TP53* result in cancer with very high penetrance [6, 7].

Seminal studies by Haapaniemi et al., [8] and Ihry et al., [9] published in 2018 identified that the activity of p53 negatively impacts the outcome in a CRISPR/Cas9 experiment and showed that transient p53 inhibition could increase the number of successfully CRISPR-modified cells. Additionally, both studies argued for the importance of monitoring the p53 functionally in CRISPR/Cas9-modified cells used for clinical purposes, as the CRISPR/Cas9 could give a selective advantage to cells with *TP53* mutations (also discussed in [10]). More specifically, in contrast to *TP53* wild-type (WT) cells, *TP53* mutated cells are not expected to leave the cell cycle or go into apoptosis following DNA damage, and would thereby be relatively enriched in a mixed population containing both WT and mutated cells, following the exposure to CRISPR/Cas9.

Considering the important implications of these findings for both clinical and experimental use of CRISPR/Cas9, we set out to further expand our understanding of the p53 pathway in this context. In a recently concluded study [11], we could confirm that CRISPR/Cas9, as well as other p53-activating interventions (Etoposide, AMG232, and hypoxia), gives cells with inactivating mutations in p53 a selective advantage in a mixed population, resulting in the enrichment of the mutated cells following treatment. As many proteins acting up- and downstream of p53 are identified as important tumor suppressors, we further explored if additional members of the p53 interactome play a non-redundant role in the CRISPR setting. To this end, we performed CRISPR screen experiments and analyzed full genome CRISPR screen data of more than 800 human cancer cell lines in the DepMap portal (https://depmap.org/portal/). Combining the results of these approaches identified an extended universe of genes playing an important role in the cellular response to CRISPR/Cas9. In Figure 1A, a list of genes is shown with the top correlations to *TP53* sgRNA enrichment found in the DepMap datasets. A strong positive correlation indicates that sgRNAs targeting the gene have a similar effect as sgRNAs targeting *TP53.* In other words, cells with inactivating mutations in the identified genes are expected to be enriched in response to CRISPR/Cas9 exposure in a p53 dependent manner. These genes are, thus, net positive regulators of the p53 pathway (e.g., CHEK2 and ATM, well known to activate p53 following dsDNA damage). In contrast, a strong negative correlation indicates genes that are net negative regulators of the p53 pathway (e.g., MDM2 and MDM4, well known to suppress the activity of p53). Further analysis showed that the identified proteins were characterized by a high degree of physical interactions (Figure 1B). Importantly, inactivating mutations or silencing of many of the positive correlating genes (*TP53BP1, CDKN1A, USP28, CHEK2, ATM, XPO7, UBE2K,* see [11] for links) have been identified in many different cancers. Similarly, activating mutations, copy number amplifications, and overexpression have been identified in various cancer for many of the negatively correlating genes (*MDM2*, *PPM1D*, *MDM4*, *PPM1G*, *WDR89*, *USP7*, *DDX31*, *TERF1*, see [11] for links). We could also show that transient p53 inhibition, using a combination of p53 siRNAs, could abrogate the CRISPR/Cas9-mediated enrichment of cells with mutations in e.g. *TP53*, *CHEK2,* and *CDKN1A*.

**Figure 1:**
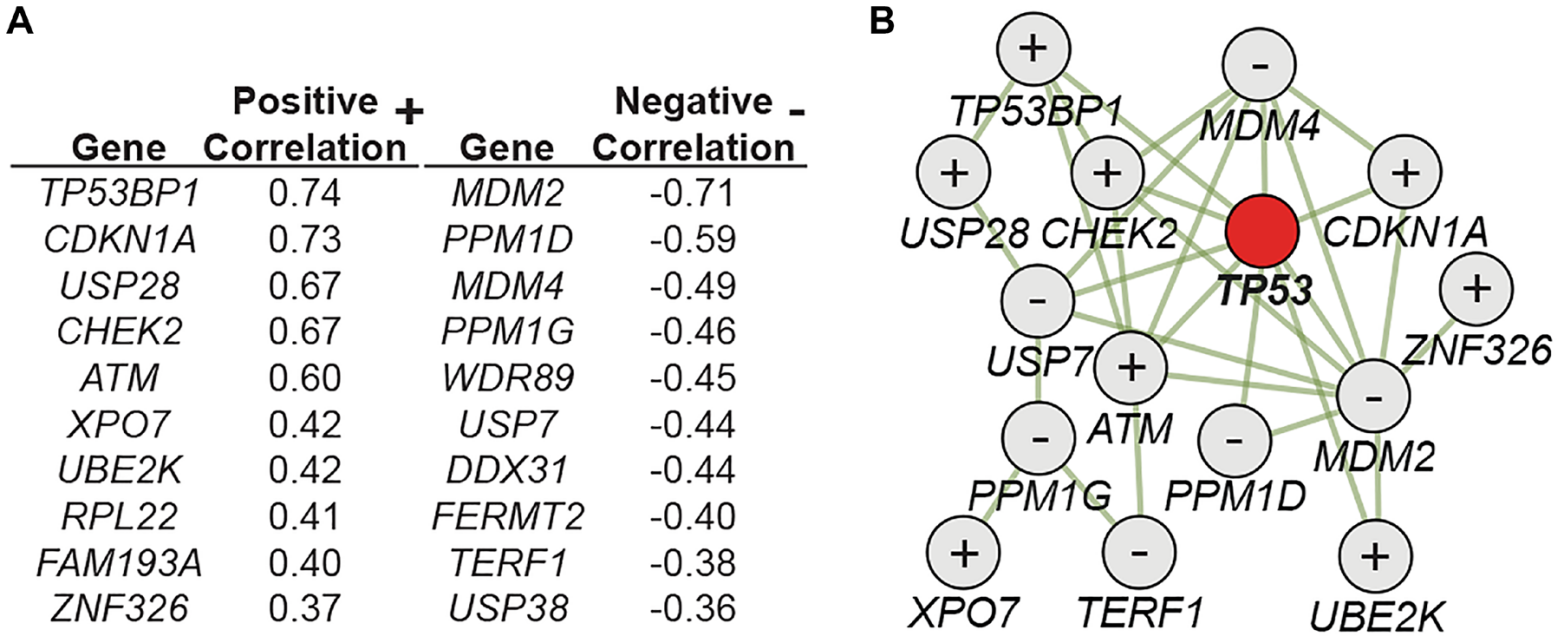
A CRISPR-p53 interactome. (**A**) Correlation scores comparing the enrichment/depletion of sgRNAs targeting indicated genes to sgRNAs targeting *TP53* in full genome CRISPR screens (*n* = 808). (**B**) Interaction map showing physical interactions between identified proteins, as well as the net functional role (+ or −) as specified in (A). Figure adapted from Jiang et al., Cancer res., 2022 with permission.

In conclusion, our data support that the identified p53-CRISPR interactome (Figure 1) should be monitored for mutations in the clinical CRISPR/Cas9 setting, as cells with mutations in these genes, plausibly linked to cancer development, could be enriched by CRISPR/Cas9. Additionally, transient p53 inhibition could be considered to not only make CRISPR/Cas9 more efficient [8, 9], but also to decrease the enrichment of cells with mutations in the CRISPR-p53 interactome.
